# Fractal Dimension of Basalt Fiber Reinforced Concrete (BFRC) and Its Correlations to Pore Structure, Strength and Shrinkage

**DOI:** 10.3390/ma13143238

**Published:** 2020-07-21

**Authors:** Yue Li, Aiqin Shen, Hua Wu

**Affiliations:** Key Laboratory for Special Region Highway Engineering of Ministry of Education, Chang’an University, Xi’an 710064, China; kkbosshua@163.com

**Keywords:** basalt fiber, fractal dimension, strength, correlation, grey correlation theory

## Abstract

In this study, we focused on exploring the correlations between the pore surface fractal dimensions and the pore structure parameters, strength and shrinkage properties of basalt fiber-reinforced concrete (BFRC). The pore structure of BFRCs with various fiber contents and fiber lengths was investigated using mercury intrusion porosimetry (MIP) measurements. Through Zhang’s model, the fractal characteristics of BFRCs in the whole pore size range and in different pore size ranges were calculated from the MIP test data. The results showed that the addition of BF increased the total porosity, total pore volume and pore area but decreased the average pore diameter, indicating that BFs refined the pore structure of the concrete. BFRC presented obvious fractal characteristics in the entire pore-size range and individual pore-size ranges; generally, the fractal dimension increased with increasing fiber content. Moreover, correlation analysis suggested that the fractal dimension of BFRC in the whole pore-size range (FD) was closely related to the fractal dimension in the macropore region (D_m_) and average pore diameter (APD). The influence of pore structure factors on mechanical strength and shrinkage was studied by grey correlation theory, and the results showed that D_m_ showed positive correlations with strength and fracture energy, with increasing D_m_ tending to strengthen and toughen the concrete. An increase in fiber content and length was detrimental to reducing the drying shrinkage strain. In the transition pore region, the fractal dimension (D_t_) at diameters ranging from 20 to 50 nm and shrinkage strain exhibited a highly linear relation. These results merit careful consideration in macro-property evaluation by using the pore surface fractal dimension in a specific region instead of the whole region. Finally, grey target theory was applied to evaluate the rank of the mechanical strength and shrinkage of concrete, and the results showed that the overall properties of concrete with a BF length of 18 mm and a BF content of 0.06% ranked the best.

## 1. Introduction

Concrete is a brittle material with low tensile strength. Concrete structures are exposed to the natural environment and subjected to the coupling effect of environmental factors and load during their whole service life. Shrinkage-induced cracks spread and expand rapidly under repeated loading, which significantly lowers the load-bearing capability and fatigue performance of concrete [1]. Thus, structural damage can occur once the ultimate tensile strength of concrete is reached, which can result in a durability issue.

To reduce crack sensitivity and solve the limitation of quasi-brittle fractures in concrete, the utilization of fiber in concrete has attracted extensive attention in recent years. Fiber-reinforced concrete (FRC) has been developed into a new type of concrete that has been successfully applied in engineering. The main reason can be attributed to its excellent properties, such as tensile strength, crack and shrinkage resistance, and fracture toughness, which also substantially improves the durability of concrete. Researchers have engaged in extensive efforts to study the effects of different types of fibers on cracking, shrinkage resistance and toughness properties. Steel fiber and polypropylene fiber are reinforcement materials that are highly valued by researchers because of their outstanding cracking resistance and enhanced tensile strength in concrete [2,3,4,5,6,7].

Basalt fibers are made by melting and drawing basalt rocks; there are no harmful gases emitted during the whole manufacturing process, which is why basalt fiber is often called “green fiber”. Owing to its apparent advantages, such as environmental friendliness, cost effectiveness, light weight, high modulus, and excellent thermal and chemical stability [8,9,10,11,12], basalt fiber has recently been considered an alternative to other fibers. Previous studies on basalt fiber reinforced concrete (BFRC) paved a solid foundation in understanding the effect of basalt fiber length and dosage on the strength [13], fracture properties [14,15] and shrinkage performance [16,17] of concrete. It has been acknowledged from the aforementioned investigations that basalt fiber distinctly improved the anti-cracking and load-bearing ability of concrete. It is also well known that the strength, durability and shrinkage performance of concrete are mainly affected by pore structure. Generally, the pores in concrete are categorized into gel pores (d < 10 nm), transition pores (10 nm < d < 100 nm), capillary pores (100 nm < d < 1000 nm) and macropores (d > 1000 nm). For instance, the porosity of capillary pores is considered nondetrimental to the chloride diffusivity of concrete, while there are good positive correlations between the gas permeability and total porosity of transition pores and capillary pores [18]. The gel pores and capillary pores are beneficial for strength [19]; nevertheless, drying shrinkage strain is essentially influenced by transition pores with diameters smaller than 50 nm [20].

The actual pore structures of concrete are complex and have extremely irregular morphologies; therefore, some researchers believe that the pore structure cannot be wholly characterized by using traditional parameters such as porosity, average pore diameter, and pore volume [21,22]. Fractal theory can effectively qualify the irregularity and complexity of the pore structure and has been intensively applied in characterizing the topography of cementitious materials. Liu et al. [23] calculated the pore surface fractal dimension of cement paste based on mercury intrusion porosimetry (MIP) test data and reported that pore structures with various pore sizes have different fractal characteristics. Cui et al. [22] investigated the pore surface fractal characteristics of concrete reinforced with three types of fibers by using two typical models: the Menger sponge model and Zhang’s model. They reported that uniform fractal characteristics within the whole pore size range could be obtained through Zhang’s model. By using Friesen and Mikula’s model, Ji’s model, Usteri’s model and Pia’s model, Niu et al. [24] established a three-dimensional intermingled fractal unit (3D-IFU) model to qualify the fractal characteristics of fiber-reinforced concrete with various fiber contents. The results showed that the pore structure characteristics depended on the matrix strength and fiber content. Since the fractal dimension can effectively qualify the complexity of pore structures, it must be closely related to the mechanical properties and permeability of concrete, which can be found in the literature [21,25,26,27].

The addition of fibers to the concrete inevitably introduces pores and increases the interface area, which may simultaneously change the fractal characteristics of pore structures. As mentioned before, many studies have been performed to investigate the mechanical properties, fracture performance, shrinkage and durability of fiber-reinforced concrete. Some studies have also been conducted to explore how basalt fiber affects the pore structure parameter and have further established the simple relationship between porosity, average pore size and macro properties [28,29,30]. However, there is little research concerning the fractal characteristics of the pore structure of BFRC. The object of this study is to investigate the pore structure and fractal characteristics of BFRCs with various fiber lengths and contents and to determine the correlation between the fractal dimension and the pore structure factors, mechanical strength and shrinkage of BFRCs. The pore parameters, such as porosity and pore size distribution, were obtained from an MIP test, and the pore surface fractal dimension in whole pore size ranges and in different pore size regions of BFRCs were calculated accordingly. A grey relational analysis was conducted to investigate the precedence among pore structure factors that affect the strength and shrinkage of BFRCs. Finally, the approaching degree to optimal comprehensive performance of BFRCs was evaluated and ranked by grey target analysis.

## 2. Experimental Study

### 2.1. Materials and Mix Design

**Figure 1:**
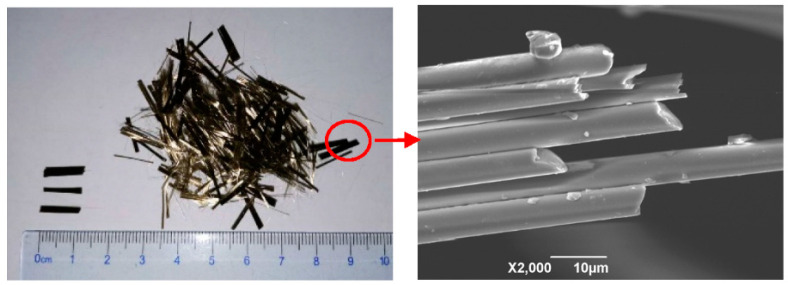
Geometry and SEM micrographs of basalt fibers.

Basalt fiber reinforced concrete (BFRC) was prepared by the following ingredients: Grade 42.5 ordinary Portland cement (Yaobo Co., Xi’an, China) with the density of 3011 kg/m^3^, two sizes of crushed stone (maximum size of aggregate of 9.5 mm and 19 mm) (Chuangqi Co., Weinan, China), river sand (Chuangqi Co., Weinan, China) with fineness modulus of 2.6, superplasticizer (QINF Co. Weinan, China) with 26% water reduction rate, water, and basalt fiber (Shijin Co., Dongyang, China). The concrete mix proportion of concrete can be found in Table 1, the water/binder ratio (W/B) was 0.36, the designation of BFRC and mass proportions were given in Table 2 and Table 3, respectively. The properties of basalt fiber adopted in this experiment were given in Table 4. It should be noted that to achieve the target slump (120–140 mm), superplasticizer content used in BFRC mixture is slightly higher than that of in control concrete mixture. As shown in Table 1, the content of superplasticizer for control concrete is 4.83 kg/m^3^. For BFRCs, the content of superplasticizer is 0.0125 of the mass of cement, which is 5.49 kg/m^3^. It should be mentioned that all mixture had achieved the target slump. Figure 1 presents the geometry and microstructure of basalt fiber under the scanning electron microscope (SEM) (Hitachi Co., Tokyo, Japan).

### 2.2. Specimen Preparation

**Figure 2:**
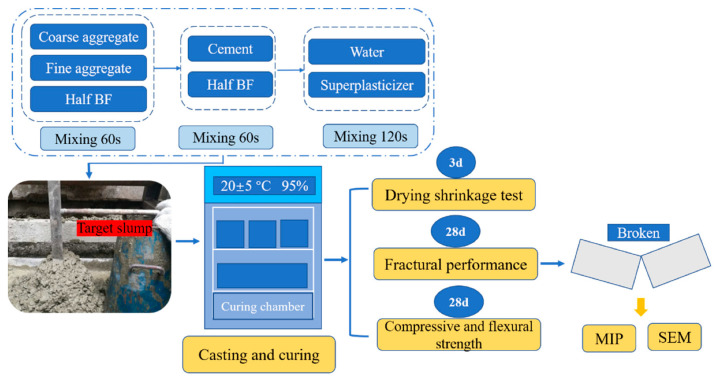
The diagram of test program.

All solid ingredients including sand, coarse aggregate and half mass of basalt fiber were first mixed for 60 s in forced concrete mixer (Keda Co., Cangzhou, China), then pour out rest of basalt fiber and cement into a blender and remixed for 60 s. The next step was to add water and water reducer, and then, the mixture was remixed for 120 s. Casted the mixture into molds and vibrated on a high-frequency vibration table. Finally, moved specimens into environment room with the condition of 20 ± 5 °C temperature and 95% humidity for curing. The diagram of test program is given in Figure 2.

### 2.3. Test Method

#### 2.3.1. Compressive Strength

Control and BFRCs specimens were prepared according with Chinese standard GB/T50081-2002. Cube specimens of 150 × 150 × 150 mm^3^ were used for compressive strength test after curing 28 days. Each series of BFRCs had three specimens. The compressive strength test was loaded through a 5000 kN electro-hydraulic servo pressure testing machine (Suns Co., Shenzhen, China), and the compressive strength was calculated by Equation (1).

f_c_ = F/A (1)

where *f_c_* means compressive strength (MPa), *F* represents for ultimate load (N), *A* is the bearings area (mm^2^).

#### 2.3.2. Flexural Strength

Chinese standard GB/T50081-2002 also was adopted for flexural strength test. According to standard, prisms specimens with size of 100 × 100 × 400 mm^3^ were prepared, tests were conducted using a YES-300B tester (Suns Co., Shenzhen, China) with 0.05 MPa/s to 0.08 MPa/s loading rate. Each series of BFRCs had three specimens. The calculation of flexural strength of concrete is according to Equation (2), which listed as follows:

F_f_ = FL/bh^2^ × 0.85 (2)

where *F_f_* means flexural strength (MPa), *F* represents for ultimate load (N), *L* is the distance between supports (mm), *b* and *h* are the width and height of concrete specimens (mm), respectively. In this study, *L* is set as 300 mm, and both *b* and *h* are 100 mm.

#### 2.3.3. Pores Structure

In this study, MIP was adopted to determine the pore size distribution of the control concrete and BFRCs. After curing 28 days, one prims specimen with 5 mm height of each concrete was selected, then take out a piece of specimens with a size of 10 mm × 10 mm × 5 mm for MIP test. To ensure the accuracy of the data, the aggregate part should be avoided. The pore structure tests were carried out by using an Autopore IV 9500 mercury porosimeter produced by Micromeritics Company, Norcross, Georgia, America.

#### 2.3.4. Fracture Performance

**Figure 3:**
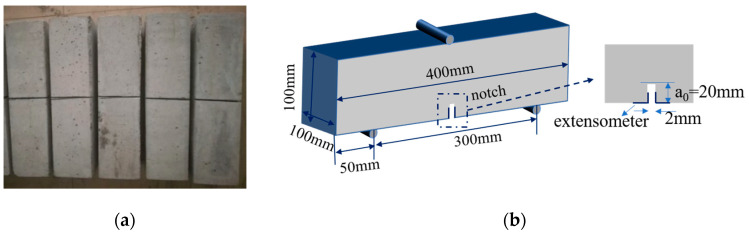
Fracture properties test of BFRCs. (**a**) fracture specimens; (**b**) test configuration.

Twenty-one notched beams with size of 100 × 100 × 400 mm^3^ were tested by a three-point bending test (Suns Co., Shenzhen, China) based on The International Union of Laboratories and Experts in Construction Materials, Systems and Structure (RILEM), control concrete and each series of BFRCs has three specimens. A notch with a depth of 20 mm and a thickness of 2 mm was made in the midspan, as shown in Figure 3. The crack mouth opening displacement (CMOD) corresponding to the applied load was measured by the clip-on extensometer. The tests were carried out on a universal testing machine with a maximum capacity of 100 kN, and the loading rate was 0.02 mm/min. The fracture energy (*G_f_*) was determined by using the following formula (Equation (3)) given by RILEM 50-FMC Technical Committee.

(3) $$G_{f}=\frac{W_{0}+{\mathrm{mg}\delta }_{0}}{b\left(h-a_{0}\right)}$$

where *W*_0_ is the area under the load-CMOD curve (N/mm), *mg* is the self-weight of the specimen (kg), *δ*_0_ is the maximum mid-span displacement (mm), *b* and *h* are the width and height of the beam (mm), respectively. *a*_0_ is the height of notch (mm).

#### 2.3.5. Drying Shrinkage

Three 100 × 100 × 400 mm^3^ prisms of each BFRC were prepared for free drying shrinkage test (Monitor Instrument Co., Shenzhen, China).

**Figure 4:**
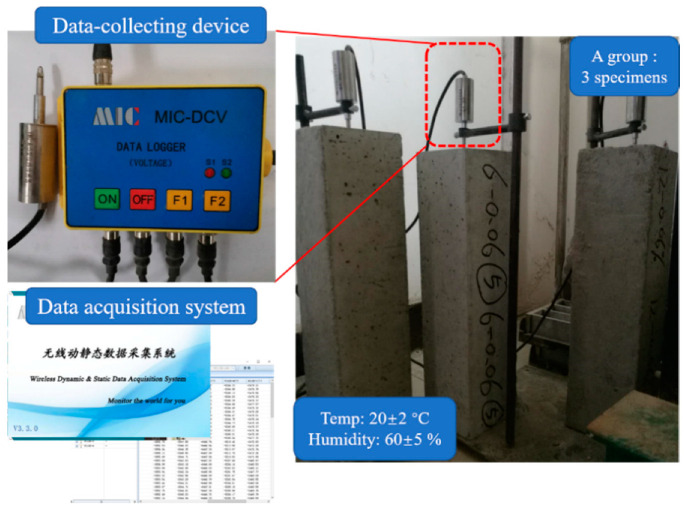
Free shrinkage test equipment.

For continuous monitoring the shrinkage strain of concrete as curing time increase, voltage data collector was matched with displacement meter, as can be seen in Figure 4, were used for data measurement and collection. After curing 3 days, settled specimens in condition of 20 ± 2 °C temperature and 60 ± 5% humidity for shrinkage test, as shown in Figure 4. Each series of BFRCs had three specimens.

#### 2.3.6. SEM

The cube test samples were taken from the specimens after strength test with size of 1 cm^3^. Before the SEM test, cube samples need to be dried, dusted, and the gold sprayed on their surface. We took three pieces of samples from each series of concrete for the SEM analysis.

## 3. Results and Discussion

### 3.1. Pore Structure

#### 3.1.1. Pore Size Distribution

**Figure 5:**
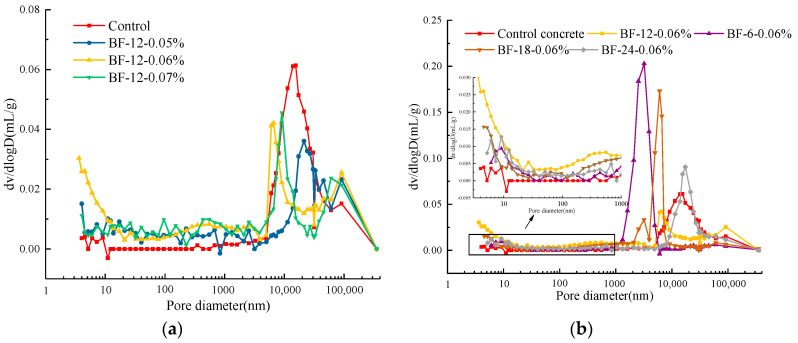
Pore size distribution of BFRCs with different (**a**) BF content (**b**) BF length.

An MIP test was carried out to investigate the pore structure of concrete with various contents and lengths of BF. The pore size distribution curves of each BFRC sample and the control concrete are shown in Figure 5. The highest value of the curve represents the highest proportion of pore volume in the concrete, which is also called the most likely aperture. In Figure 5a, it can be seen that the addition BF-12-0.06% and BF-12-0.07% moves the peak value to the left, meaning that it lowers the most likely aperture value. Comparing the cumulative pore volume of control concrete and BFRCs at pore sizes ranging from 10 to 1000 nm, it can be observed that the addition of BF increases these micropore contents. As the BF content increases from 0.05% to 0.06%, the peak value in the range of 5000 to 500,000 nm gradually moves to the left. These attained results clearly suggest that the addition of BF decreased the coarseness of the pore size and refined the pore structures. A similar trend was also reported by Zhang et al. [18], who also found that concrete with BF presented higher microporosity than base concrete. However, there existed a peak value where the pore size was nearly 100,000 nm, and the porosity of the BFRCs was higher than that of the control sample. The reason could be ascribed into two parts. Firstly, the addition of fiber may introduce a large number of bleeding channels or internal cracks during the hardening process of concrete [31]. Secondly, the samples which are prepared for the MIP test are obtained by cutting, which may change the pore structure [18]. Moreover, we can see that the peak appears in 100,000 nm is much smaller than that which appears around at 10,000 nm, indicating small amounts of pores with a size of 100,000 nm, therefore, the refined effect of fibers on the pore structure of concrete could also be concluded.

The behavior trend of pore size distribution of the control and BFRCs with different BF lengths are depicted in Figure 5b, from the curve, the most probable aperture of concrete can be obtained. It is interesting to note that BF length imposes a more obvious impact on the concrete pore size distribution curve in this study. At the same BF content, concrete with 6 mm BF reveals the smallest most likely aperture value, followed by BF-18-0.06%, BF-12-0.06% and BF-24-0.06%. The most likely aperture value of BF-24-0.06% is slightly higher than that of the control concrete. This can be attributed to two main reasons: a decrease in the workability of the BFRCs, which inevitably draw into voids in the concrete matrix, and the bridge effect of fibers, which facilitates the interconnection of pores along the length of the fibers [32,33].

**Figure 6:**
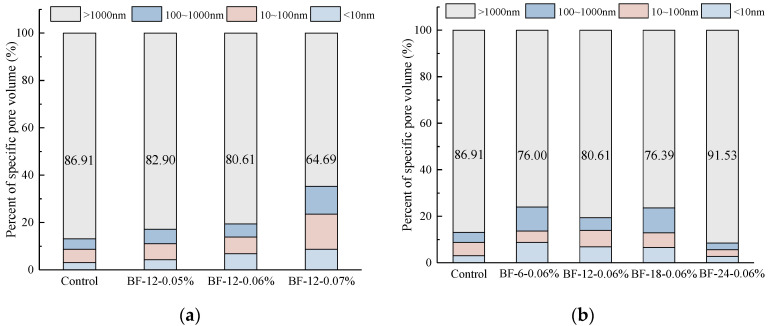
Percent of specific pore volume of BFRCs with different (**a**) BF content; (**b**) BF length.

The percentage of specific BFRC pore volume is given in Figure 6. In Figure 6a, the addition of BF increases the volume of pores with diameters less than 1000 nm, especially in BF-12-0.07%, and the volume of gel pores, transition pores and capillary pores increased significantly when compared with the control concrete. The reason may be that BFs connect large pores and increase the interface area, which results in an increase in the small pore volume. Figure 6b presents the pore volume of BFRCs with various fiber lengths. It can be seen that with increasing fiber length, the volume of gel pores decreases obviously. For BF-24-0.06%, the volume of pores with diameters less than 1000 nm is lower than that in the control concrete.

#### 3.1.2. Porosity

**Figure 7:**
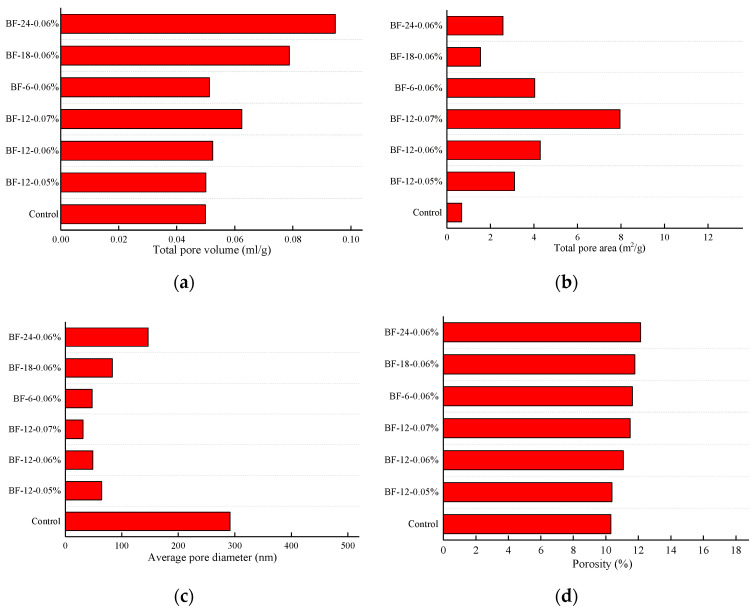
Pore structure parameter of BFRCs. (**a**) Total pore volume; (**b**) Total pore area; (**c**) Average pore diameter; (**d**) Porosity.

**Figure 8:**
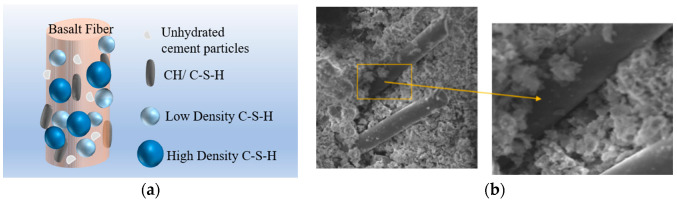
Diagram of BF-concrete interface. (**a**) Schematic of BF-concrete interface; (**b**) Image of BF-concrete interface.

Figure 7 presents the pore structure parameters of the control concrete and BFRCs. From the Figure 7, the addition of BF has an evident impact on the pore structure of concrete. As the BF content increases, the total pore volume, total pore area and porosity all show increasing trends, whereas the average pore diameter decreases. This indicates that the pore structure is refined by BF, and the effectiveness of BF in refining the pore structure of concrete increases with increasing BF content. The increase in porosity may be attributed to the fact that adding BF increases the concrete interface. The diagram of the interface between the BF and concrete is given in Figure 8. As presented in Figure 8a, BF is hydrophilic and results in more hydration products adhering to its surface [34]. However, this attachment is not completely tight, and there are small pores in the interface between the fibers and the concrete, which can be clearly seen in Figure 8b. Therefore, the higher the fiber content is, the higher the porosity level.

The trends of variations in the pore structure with the BF length are not similar to those in concrete with different BF contents. With increasing BF length, the porosity of the BFRCs first decreases and then increases. As expected, the porosity of BF-24-0.06% is higher than that of other BFRCs with the same fiber content. Notably, BF-24-0.06% also shows a larger average pore size, which is approximately 3 times higher than that of BF-12-0.06%. Combined with the pore size distribution, it can be deduced that refinement of concrete pore structure is weakened when BF with a length of 24 mm is added.

#### 3.1.3. Fractal Dimension of Pore Surface

To quantitatively assess the parameter for the pore size distribution of concrete matrices by BF, the fractal dimensions of the pore surfaces were calculated based on the thermodynamic method proposed by Zhang et al. [35,36]. The expression is given as follows.

(4) $$\ln\left(\frac{W_{n}}{r_{n}^{2}}\right)\;{=D}_{s}{\mathrm{lnQ}}_{n}^{\prime }+\mathrm{lnC}$$

where *W_n_* is the cumulative intrusion work, W_n_ = $\Sigma_{i=1}^{n}{=P}_{i}{\Delta V}_{i}$, and *P_i_* (psia) and *V_i_* (mL/g) are the mercury intrusion pressure and the intrusion volume at the ith intrusion of mercury; $Q_{n}^{\prime }=\frac{\sqrt[{3}]{V_{n}}}{r_{n}}$, *V_n_* (mL/g) is the accumulative intrusion volume, *r_n_* (nm) is the pore diameter, and *C* is the model constant. According to Equation (4), after calculating $\frac{W_{n}}{r_{n}^{2}}$ and $Q_{n}^{\prime }$, $\frac{W_{n}}{r_{n}^{2}}$ is plotted versus $Q_{n}^{\prime }$ in log-log coordinates. *D_s_* is determined as the slope value of the fitting line.

**Figure 9:**
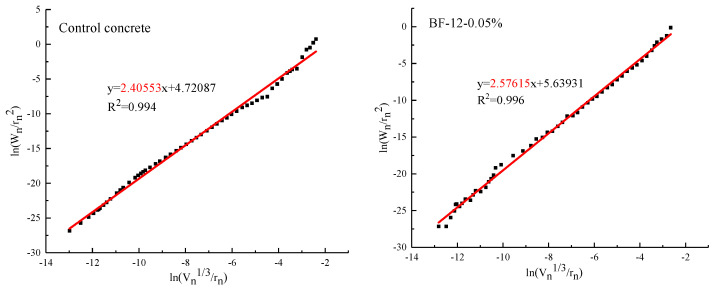

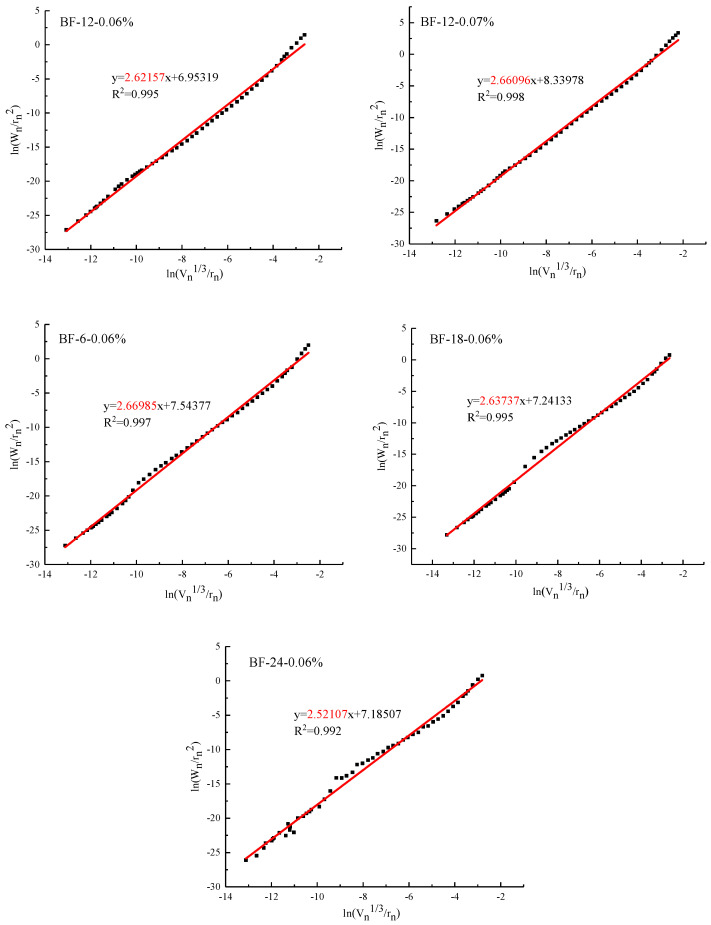
Calculation results of pore surface fractal dimension of BFRCs.

The results of the surface fractal dimension (FD) of different BFRCs are presented in Figure 9. The FD of BFRCs ranges from 2.52 to 2.66, which is higher than that of the control concrete (2.40), indicating that BF increases the irregularity of the pore fractured surfaces. Based on the analytical outcome of the pore size distribution and the pore parameters in Figure 5 and Figure 7, BF refines the pore structure of concrete and transforms large pores to small pores. An increased number of smaller pores leads to the pore structure of concrete becoming more dispersed, which affects the increase in fractal dimension [37]. A primary observation is that FD increases with increased BF content, while it generally decreases as BF length increases. From the pore size distribution, gel pores and capillary pores increase significantly as the fiber content increases, which results in an increase in FD. However, with increasing fiber length, the gel pores decrease, and the average pore diameter increases, causing a decrease in FD. The reduction in FD in BF-24-0.06% is considered to be due to the increment in large pores, which reduces the complexity of surface topography.

**Figure 10:**
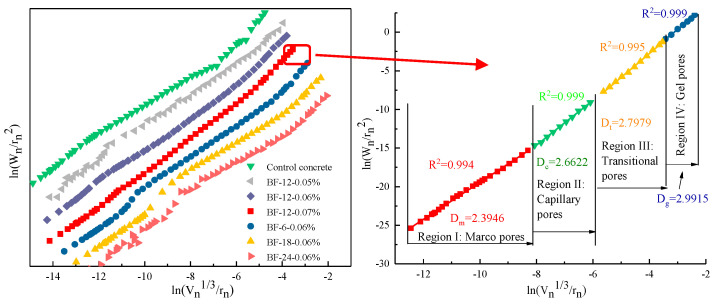
Calculation of fractal dimension of BF-6-0.06% in four regions.

Moreover, by using Zhang’s model, Kim et al. [36], Zhu et al. [38] and Li et al. [26] also determined the fractal characteristics of cementitious-based materials with various pore diameters. Similarly, the fractal dimension of the pore surface was evaluated in different regions, as shown in Figure 10. Pores are divided into four specific regions. Thus, the FD of each region is calculated, and the results are given in Table 5. In general, the pore structure of BFRCs presents significant fractal characteristics in four regions, and the fractal dimension follows D_m_ < D_c_ < D_t_ <D_g_, indicating that the smaller the pore size is, the more complex the pores are. In Regions I and II, D_m_ and D_c_ of the control concrete are smaller than those of the BFRCs. However, in Regions III and IV, the D_t_ and D_g_ of the control concrete are higher than 3.0, which is not in conformity with the basic assumption of Zhang’s model. Some researchers attributed this phenomenon to the existence of ink-bottle pores. In the investigation of Yu et al. [39], the results indicated that many ink-bottle pores were present in pore sizes ranging from 20 to 100 nm. Xiao et al. [40] also reported that the ink-bottle effect exists in both capillary pores and gel pores. BF shifts the peak of the pore size distribution curve from right to left, decreasing the most likely aperture of concrete, and the increment of the interface area between the fiber and concrete leads to an increase in capillary pores and transition pores, which finally results in an increase in FD in Regions I, II, and III. The difference in D_g_ between BFRCs is not as obvious as D_m_ and D_c_. This can be ascribed to the fact that the properties of gel pores are closely related to the hydration products [36]. BF is an organic fiber and is not involved in the hydration process; thus, the D_g_ values of BFRCs are close to each other.

In order to obtain the interrelationship among pore parameters, correlation analysis was performed among FD, porosity with different pore size, D_m_, D_c_, average pore diameter (APD), total pore volume (TPV) and total pore area (TPA). The results are presented in Table 6.

The relationship between FD and total porosity is not significant, implying that porosity cannot represent the complexity of pore structure. FD presents a close relationship with APD and D_m_, and the correlation coefficient between FD and D_m_ is the highest. With an increase in FD, the average pore diameter decreases, indicating that the smaller the pore size is, the higher the degree of complexity for the pore structure. In this study, the most likely apertures of the control concrete and BFRCs are all macropores, suggesting that macropores are widely dispersed in the whole pore structure. This could be ascribed to the strong correlation between FD and D_m_. Moreover, it can be seen that the gel pores and capillary pores have a good correlation. The reason might be related to the fact that a similar behavior can be observed in gel pores and capillary pores with increasing fiber length and content. Another observation is that macroporosity displays an evident relationship with TPV. This makes sense, as more macropores indicate more TPV.

### 3.2. Compressive and Flexural Strength

**Figure 11:**
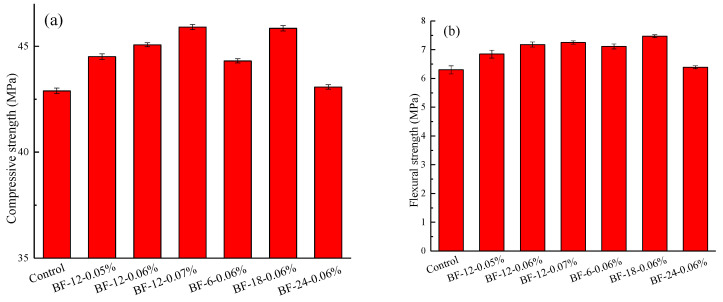
Strength of control concrete and BFRCs: (**a**) Compressive strength (**b**) Flexural strength).

The obtained compressive and flexural strengths of the specimens with four different BF lengths and contents are displayed in Figure 11. Normal concrete was used as a reference. From Figure 11a, it can be seen that the addition of BF has a trivial effect on enhancing the compressive strength of the concrete. With increasing BF content, the compressive strength of the BFRC increases. However, as the BF length increases from 6 mm to 24 mm, the compressive strength value first increases steadily and then decreases. The highest value for the compressive strength is obtained from the concrete mixture incorporated with a content of 0.06% fiber having a length of 12 mm, which indicates a 7.02% increase in compressive strength compared to the control concrete. BF-24-0.06% presents the smallest compressive strength among the BFRCs. This may be attributed to two aspects. On the one hand, long fibers are more difficult to disperse evenly than short fibers. On the other hand, voids introduced during BFRC preparation are difficult to fully eliminate through vibration due to decreased workability.

By analyzing the flexural strength data of the BFRCs in Figure 11b, it can be seen that the enhanced extent of the flexural strength of concrete is greater than the compressive strength, which also reflects the strong upgrading effect of BF on the flexural strength of concrete. This is also in accord with previous investigations [10,41]. With a constant BF length of 12 mm, the flexural strength of BFRCs increase as the fiber content increase, which implies that increasing fiber content could provide stronger anchorage and bridge effects in concrete to resist cracks. With fiber length increase, the flexural strength of BFRCs first increase and then decrease. BF-18-0.06% has the highest flexural strength among all BFRC specimens. This could be explained by the fact that randomly distributed BFs in the concrete matrix worked as micro-reinforcements to bridge cracks and toughen the concrete when subject to flexural load. The distinct drop in flexural strength in BF-24-0.06% is possibly because of its relatively poor workability. Boulekbache et al. [42] found that fiber usually aligned with an inadequate orientation when the workability of the concrete was poor, which also created a weakening zone and cracking point [32,43], finally leading to a decrease in the flexural behavior of the concrete.

### 3.3. Fracture Energy

**Figure 12:**
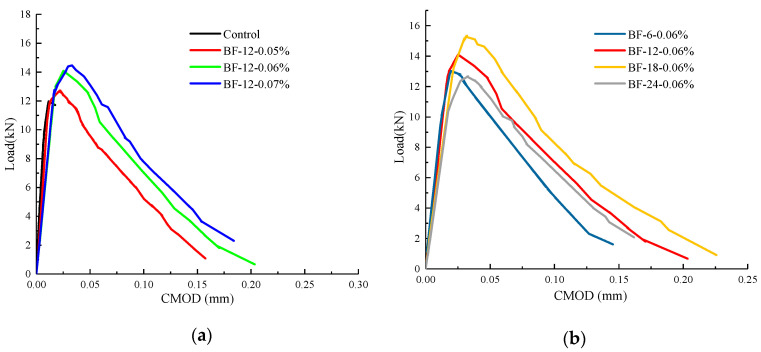
CMOD curve of BFRCs with different (**a**) BF content; (**b**) BF length.

The applied load versus CMOD curves for BFRC notched specimens are shown in Figure 12. The fracture parameters of BFRCs are presented in Table 7.

The addition of BF considerably increases the peak load, maximum deflection and fracture energy (G_f_) of the concrete. From the Figure 12a, for the control concrete, once it exceeds the peak load, the curve drops suddenly. However, the slope of the ascending part gradually decreases as the BF content increases, and the curve descends smoothly and is more stable than that of the control concrete. When constant at the same BF length, the increase in BF content enhances the fracture energy of the concrete. Similar increments were obtained by Smarzewski [44], who reported that when the basalt fiber content was within the limits of 1.5% (mass ratio), the increase in fiber content contributed to the increase in fracture energy. BF-12-0.07% possesses the highest G_f_ value compared to BFRCs with the same BF length. This value is 65.17% higher than that of the control concrete. It can be seen from Figure 12b that when the fiber length ranges from 6 mm to 18 mm, the peak load and G_f_ increase with increasing fiber length. A further increase in fiber length (24 mm) has no contribution but causes an apparent decrease in G_f_. Kabay [45] also reported that a slight decrease in fracture energy occurred when increasing the basalt fiber length from 12 mm to 24 mm. BF-12-0.06% and BF-18-0.06% show higher deflection capacities than BF-6-0.06% and BF-24-0.06%, probably because of the bridging action of basalt fiber during post-cracking.

### 3.4. Drying Shrinkage

**Figure 13:**
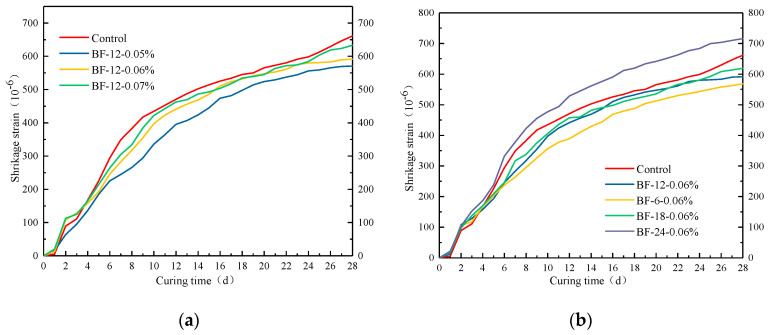
Drying shrinkage development curve of BFRCs (**a**) with different fiber content; (**b**) with different fiber length.

**Figure 14:**
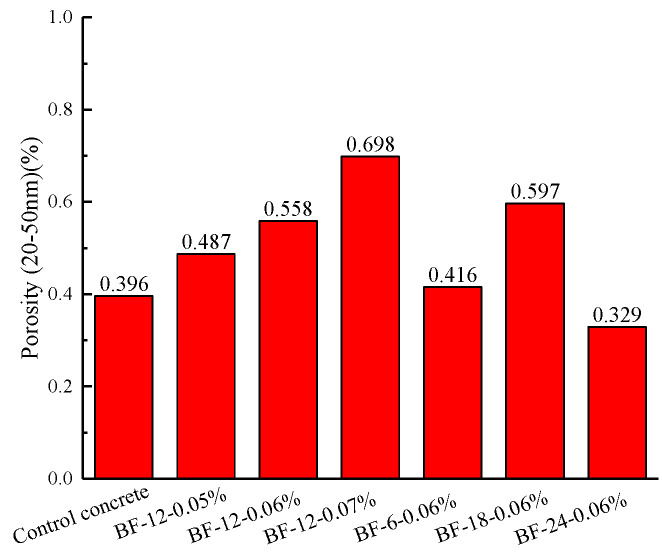
Porosity of BFRCs with pore size ranges from 20 to 50 nm.

Figure 13 illustrates the free drying shrinkage test results for the control and the BFRCs over the course of 28 days of drying. From Figure 13a, it is observed for all concrete specimens that the trend of drying shrinkage strain gradually slows down as drying time increases. Researchers have noted that the shrinkage rate reduction as the curing time increases is associated with the water loss rate. The drying shrinkage rate of the BFRCs is lower than that of the control concrete during the whole curing time. This result indicates that BF has a positive impact on reducing the drying shrinkage rate. There are three reasons for this outcome: (i) the addition of fiber improves the tensile strength of the concrete matrix, which contributes to the physically restraining shrinkage [46]; (ii) the crack development is inhibited because of the bridge action; and (iii) the shrinkage stress is transferred by the interconnections between the fibers. The maximum and lowest free drying shrinkage occurred in the control and BF-12-0.05%, respectively. The shrinkage strains of these two concrete specimens were 661.32 × 10^−6^ με and 570.81 × 10^−6^ με. By increasing the fiber content to 0.07%, the shrinkage rate of concrete is higher than that of BF-12-0.05% and BF-12-0.06%. It has also been reported that drying shrinkage is closely related to pores with diameters of 20–50 nm [47]. The porosity of small transition pores (20–50 nm) in the BFRCs is given in Figure 14. BF-12-0.07% had the highest content of small transition pores, which is the reason for the highest shrinkage strain. Sadrmomtazi et al. [48] also reported that increasing basalt fiber content resulted in increased water absorption. Based on the MIP results, as the BF content increases, the porosity of small transition pores consequently increases. These results show that increasing the fiber content may have an unfavorable effect on the reduction in the drying shrinkage rate. The shrinkage strain results of BFRCs with various BF lengths are described in Figure 13b. It has been well detected that BF-24-0.06% exhibits higher drying shrinkage than the control concrete, which increased by 8.21% compared to that of the control concrete for 28 days of moist curing time. The longer the fiber length is, the higher the shrinkage rate. This shows that an increasing fiber length has an adverse impact on decreasing the shrinkage strain. The significant increase in the shrinkage rate in BF-24-0.06% can be ascribed to its low workability, which hinders the fiber bridging effect. Ling et al. [49] found that free shrinkage strain was closely related not only to the pore size distribution but also to the weight of water loss. The amount of water loss might be associated with the morphology of the pores.

### 3.5. SEM

**Figure 15:**
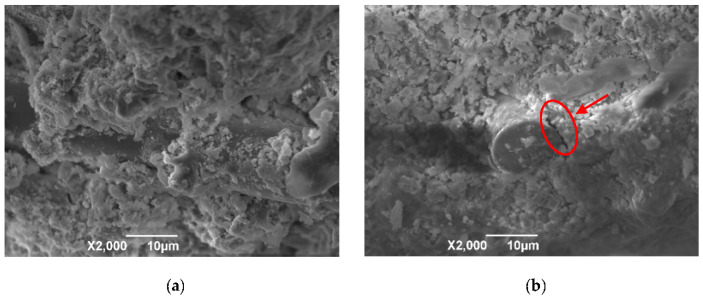

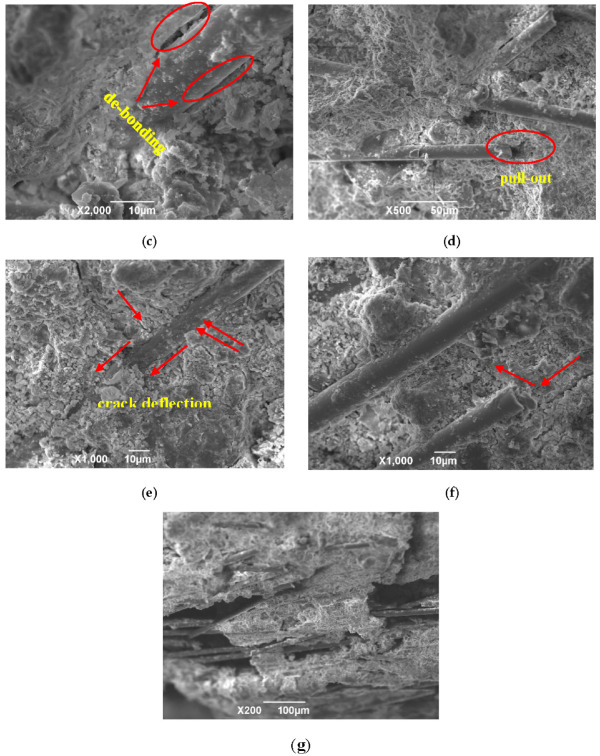
The SEM iamges of BFRCs: (**a**,**b**) show the SEM images of the BFRCs before fracture testing and (**c**–**g**) show the images of the BFRCs after fracture testing.

The microstructure of the BRRCs before and after the fracture performance test was observed though SEM, and SEM images are presented in Figure 15. Figure 15a,b show the SEM images of the BFRCs before fracture testing, and Figure 15c–g show the images of the BFRCs after fracture testing. It can be found that the surface of basalt fiber is covered with abundant cement compounds, which may include hydration products or some unhydrated cement particles, indicating a good bonding behavior between the basalt fiber and the cement matrix. This phenomenon also implies that basalt fiber and concrete work together as a whole to bear external loading. A visible void between the fiber and cement matrix is observable in Figure 15b, which is the reason why the BFRCs present higher porosity than normal concrete. When subjected to an external load, debonding between the matrix and fibers is clearer, and the area of the BF wall is partly detached from the matrix, which can be seen in Figure 15c. Figure 15d shows the reinforcement mechanism of the fiber in the cementitious matrix, in which fiber pull-out and breakage are visible and occur due to excessive shear friction [50]. As the fibers pull out of the cement matrix, the energy supplied for crack propagation is consumed by frictional stresses; therefore, the toughness of the composite could be further enhanced. This behavior is responsible for the improvement of fracture energy in the BFRCs. It can be clearly seen that the remaining part of the basalt fiber is still on the sliding surface across the cracks. In Figure 15e,f, cracks are created around the fibers and gradually develop in the matrix, and crack deflection is also observable. The fiber reinforcement mechanism is also supplemented by the contribution of limiting crack propagation, which results in the improvement of the load-bearing capability of the concrete. Figure 15g shows the image obtained from BF-24-0.06%, and basalt fiber clumping is observed. This weak distribution can be considered a reason for the decrease in strength.

### 3.6. Grey Correlation Analysis

To investigate the degree of correlation between pore structure factors and macro properties of BFRC, the grey correlation analysis was applied. The specific calculation steps are as follows:

1. The data series X_0_ = $\{X_{0}\left(1\right),X_{0}\left(2\right),\cdots ,X_{0}\left(k\right)\}$ was taken as the reference sequence, set the data series X_i_ = $\{X_{i}\left(1\right),{\;X}_{i}\left(2\right),\;\cdots X_{i}\left(k\right)\}$ as the comparison sequence.
2. Transfer the data series to the dimensionless form, the formula to normalize the data is as follows: Y_0_ = $\{Y_{0}\left(1\right),Y_{0}\left(2\right),\cdots ,Y_{0}\left(k\right)\}$, where Y_0_(k) = $\frac{X_{0}\left(k\right)}{\frac{1}{k}\sum_{i\;=1}^{k}X_{0}\left(k\right)}$, Y_i_ = $\{Y_{i}\left(1\right),{\;Y}_{i}\left(2\right),\;\cdots Y_{i}\left(k\right)\}$, where Y_i_(k) = $\frac{X_{i}\left(k\right)}{\frac{1}{k}\sum_{k\;=1}^{n}X_{i}\left(k\right)}$.
3. The correlation coefficient was obtained by the following equation: $\zeta_{i}(k)=\frac{\underset{i}{\min}\;\underset{k}{\min}\left|Y_{0}(k)-Y_{i}(k)\right|+\rho \;\underset{i}{\max}\;\underset{k}{\max}\left|Y_{0}(k)-Y_{i}(k)\right|}{\left|Y_{0}(k)-Y_{i}(k)\right|+\rho \;\underset{i}{\max}\;\underset{k}{\max}\left|Y_{0}(k)-Y_{i}(k)\right|}$, where ρ = 0.5
4. The average correlation coefficient can be calculated by the following equation: $\gamma_{i}=\frac{1}{n}\sum_{k\;=1}^{n}\xi_{i}\left(k\right)$.

**Figure 16:**
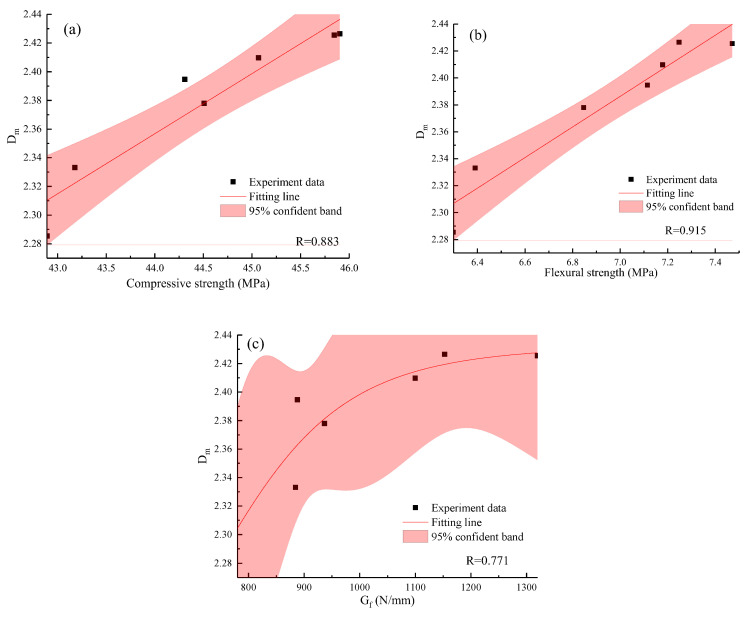
Relationship between D_m_ with (**a**) compressive strength; (**b**) flexural strength; (**c**) fracture energy.

**Figure 17:**
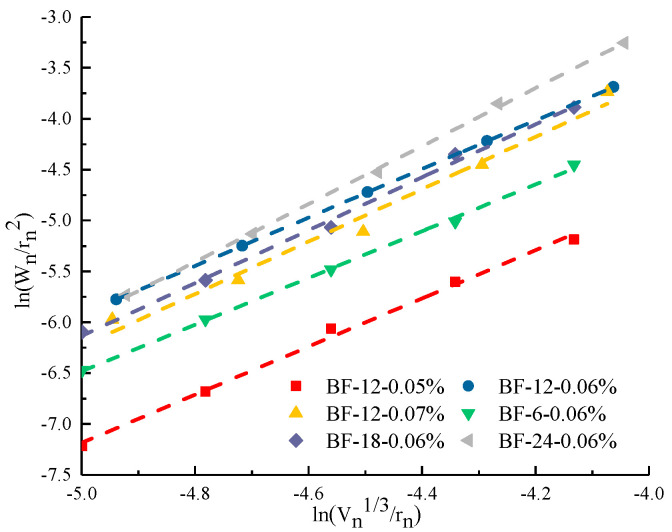
Results of D_t_ at diameter ranges 20–50 nm.

**Figure 18:**
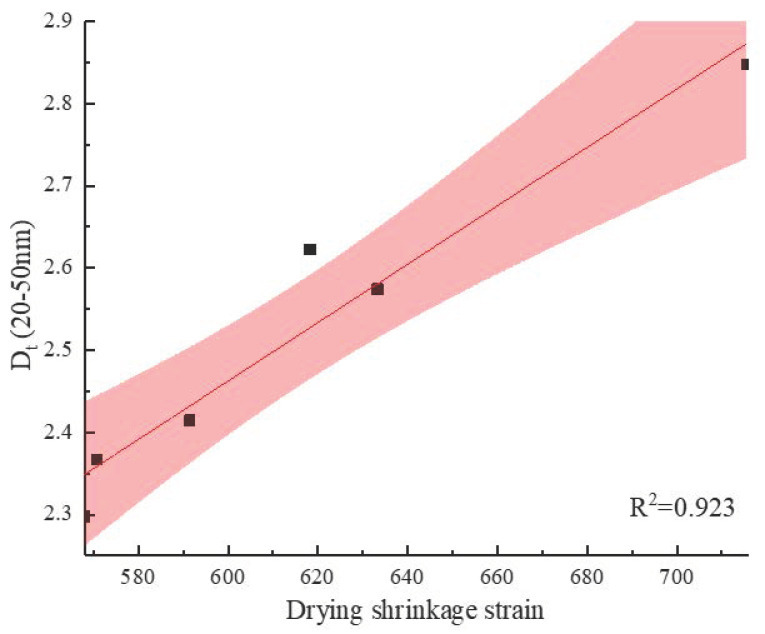
Relationship between D_t_ and shrinkage strain.

Table 8 lists the results of the average correlation coefficient (*γ_i_*). It can be seen that for flexural strength, compressive strength and fracture energy, the order of the grey correlation coefficient is D_m_ > FD > another factor. Especially in the coefficient of strength and D_m_, the *γ_i_* of D_m_ is over 0.9. From the analysis in Table 7, regression analyses are performed on our experimental data comprising the compressive strength, flexural strength, fracture energy and D_m_ of the BFRCs, as shown in Figure 16. The compressive strength, flexural strength and fracture energy of the BFRCs increase with increasing D_m_, and the correlation coefficients are relatively high. The strength is mainly determined by macropores and gel pores, and the influence of macropores is more notable. D_m_ reflects the complexity of the entire macropore; therefore, it is reasonable to find that D_m_ exerts a higher R^2^ with strength compared with FD. For drying shrinkage, the highest *γ_i_* between shrinkage strain and pore factors is the transition porosity; however, it is obviously smaller than that of *γ_i_* between D_m_ and strength. As mentioned above, the porosity of small transition pores (20–50 nm) was closely related to drying shrinkage, and the D_t_ with diameters ranging from 20 to 50 nm was calculated by using Zhang’s model, as shown in Figure 17. The relationship between D_t_ (20–50 nm) and shrinkage strain is also given in Figure 18, and good linear correlations are obtained. Generally, a high value of D_t_ is a reflection of a large transition pore content, which also means that a large water loss reduces the concrete weight. Thus, a high shrinkage strain could be expected. These results also reveal that the proposed approach may be a rational and effective way to evaluate the properties of BFRCs by using fractal dimensions calculated in different pore size ranges.

### 3.7. Grey Target Theory Analysis

**Figure 19:**
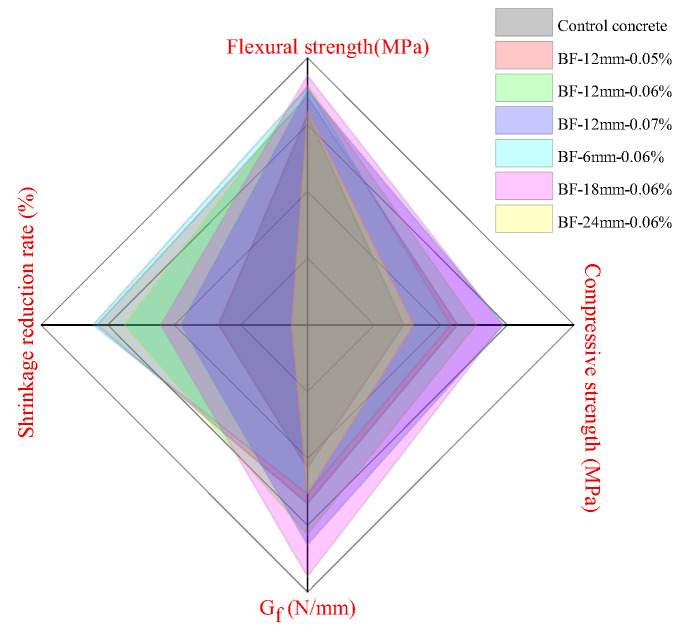
Strength, fracture energy and shrinkage reduction rate of control concrete and BFRCs.

The macro properties of the control concrete and six kinds of BFRCs are displayed in Figure 19, which clearly shows the difference in performance among all the concretes. For ease of comparison, the shrinkage reduction rate was used as a representative parameter of shrinkage properties. Thus, we can roughly compare the overall performance of the concrete by comparing the area under the curve. It can be observed that BF-24 mm-0.06% presents the smallest closed area among all BFRCs. However, the order of other BFRCs in comprehensive properties is not clear. To select the optimal fiber length and content, grey target theory analysis was used.

The specific steps are as follows:

(1) Define the index sequence as: $\omega_{k}=\left(f_{\mathrm{ck}},f_{\mathrm{fk}},F_{k},\varepsilon_{k}\right)\left(k=1,2,\cdots ,7\right)\;$; where *f_c_* is the compressive strength, f_f_ is the flexural strength, *F* is the fracture energy, *ε* is the drying shrinkage strain. 

(2) As the compressive strength, flexural strength and fracture energy are the maximum polarity, while drying shrinkage strain is the minimum polarity, the standard model is:

$$\omega_{0}\left(f_{c}\right)={\max\omega }_{k}\left(f_{c}\right),{\;\omega }_{0}\left(f_{f}\right)={\max\omega }_{k}\left(f_{f}\right),{\;\omega }_{0}\left(F\right)={\max\omega }_{k}\left(F\right),{\;\omega }_{0}\left(\varepsilon \right)={\min\omega }_{k}\left(\varepsilon \right)$$

(3) Dimensionless parametrization: $X_{0}=\left(\frac{\omega_{0}\left(f_{c}\right)}{\omega_{0}\left(f_{c}\right)},\frac{\omega_{0}\left(f_{f}\right)}{\omega_{0}\left(f_{f}\right)},\frac{\omega_{0}\left(F\right)}{\omega_{0}\left(F\right)},\frac{\omega_{0}\left(\varepsilon \right)}{\omega_{0}\left(\varepsilon \right)}\right)$ = (1,1,1,1), $X_{k}=\left(\frac{\omega_{k}\left(f_{c}\right)}{\omega_{0}\left(f_{c}\right)},\frac{\omega_{k}\left(f_{f}\right)}{\omega_{0}\left(f_{f}\right)},\frac{\omega_{k}\left(F\right)}{\omega_{0}\left(F\right)},\frac{\omega_{k}\left(\varepsilon \right)}{\omega_{0}\left(\varepsilon \right)}\right)$;

(4) The distance to target: ${∆}_{\mathrm{ok}}=\left|X_{0}-X_{k}\right|$.

**Figure 20:**
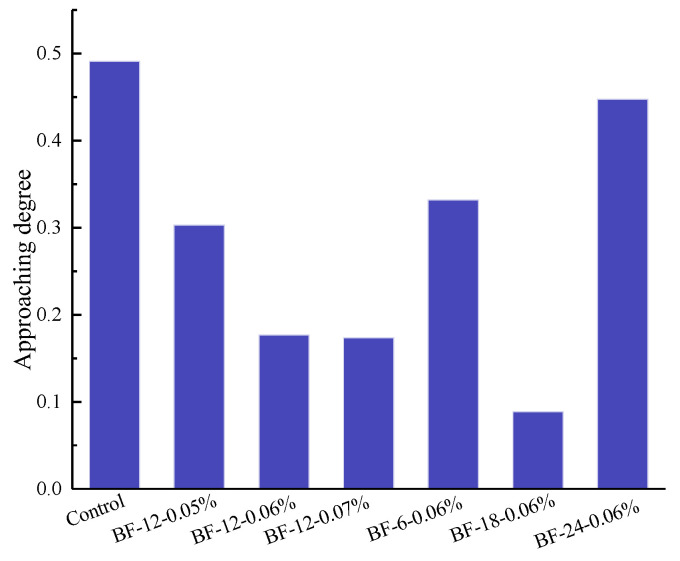
The approaching degree of concrete.

Figure 20 shows the approaching degree to the optimal properties (strength, fracture performance, shrinkage) of the control concrete and the BFRCs. The figure indicates that the closer to the center point, the better the concrete performance is. The properties of BF-18 mm-0.06% rank first, followed by BF-12 mm-0.07% and BF-12 mm-0.06%, and BF-24 mm-0.06% came last among the BFRCs. Therefore, concrete with 18 mm BF length and a BF content of 0.06% exhibits the best comprehensive performance among all the BFRCs.

## 4. Conclusions

In this experimental study, the pore structure of basalt fiber reinforced concrete (BFRC) with various fiber lengths and contents was characterized by the MIP test, and the pore surface fractal dimensions of the BFRCs in the whole pore size range and in different pore size ranges were calculated using Zhang’s model. The relationship between the pore structure parameters and the fractal dimension was analyzed. In addition, the effects of fiber content and fiber length on strength, fracture properties and shrinkage (BFRC) were evaluated, and their correlations with surface fractal dimensions were also evaluated. The following conclusions have been drawn:

1. The addition of basalt fiber (BF) decreased the coarseness of the pore size and refined the pore structures. As the fiber content increased, the total porosity and total pore volume increased, whereas the average pore diameter decreased. When the fiber length ranges from 6 mm to 18 mm, as the fiber length increases, these parameters present the same tendency. BFs diminished the macropore content, and the more BFs contained in the sample, the fewer macropores in the concrete.
2. BFRCs presented obvious fractal characteristics in the whole pore-size range; compared with control concrete, BFRCs increased the fractal dimension, indicating that the addition of BF increased the complexity of the surface topography. Moreover, the fractal analysis of BFRCs in four pore size ranges showed that BFRCs exhibited obvious fractal characteristics in every individual region. The larger the pore size is, the larger the fractal dimension. In the gel pore region, the D_g_ (fractal dimension calculated in the gel pore region) of the control concrete exceeded 3.0.
3. Through correlation analysis, the fractal dimension in the entire pore-size range (FD) was closely related to the D_m_ and the average pore diameter (APD). D_m_ presented a positive correlation with FD, while APD exerted a negative correlation with FD.
4. The presence of basalt fibers increased the flexural strength of plain concrete, while compressive strength showed no significant growth. As the fiber content increases, the flexural strength increases. Compared with the plain concrete, the compressive and flexural strength of concrete reinforced by 18 mm long BFs increased by 6.89% and 18.62%, respectively. When the fiber length was increased to 24 mm, an obvious drop in flexural strength was observed. The addition of BFs could remarkably increase the fracture energy of concrete; as the BF dosage increased, the peak load value and displacement increased. A significant drop in peak load and fracture energy was observed in BF-24-0.06% due to its uneven fiber distribution.
5. Grey correlation analysis was applied to indicate the precedence among pore structure factors in affecting the mechanical strength and shrinkage of the concrete. The results showed that D_m_ presented a stronger correlation with strength and fracture energy (G_f_) than other pore factors, and the relationship between drying shrinkage strain and fractal dimension in the small transition pore region (20–50 nm) presented a highly positive linear function. Thus, D_m_ and D_t_ could be addressed as representative pore structure parameters to predict the strength and drying shrinkage of BFRC.
6. From the evaluation result with grey target theory, the overall properties of BF-18-0.06% ranked the best, followed by BF-12-0.07% and BF-12-0.06% in sequence.

## Figures and Tables

**Table 1:** Concrete mix proportion.

Grade	W/B	Mixture Composition (kg/m^3^)				
		Cement	Water	Coarse Aggregate	Sand	Superplasticizer
C40	0.36	439	158	1136	697	4.83

**Table 2:** Fiber length and dosages.

Designation	Fiber Length (mm)	Dosage	
		Volume (%)	kg/m^3^
Control concrete	-	0	0
BF-12-0.05%	12	0.05	1.3
BF-12-0.06%	12	0.06	1.6
BF-12-0.07%	12	0.07	1.9
BF-6-0.06%	6	0.06	1.6
BF-18-0.06%	18	0.06	1.6
BF-24-0.06%	24	0.06	1.6

**Table 3:** Mass proportions of basalt fiber reinforced concrete (BFRC) mixes used.

Designation	Cement	Water	Fine Aggregate	Coarse Aggregate	BF	Superplasticizer
Control	1	0.36	1.59	2.59	0	0.011
BFRC	1	0.36	1.59	2.59	various	0.0125

**Table 4:** The physical and mechanical properties of basalt fiber.

Fiber	Density (kg/m^3^)	Tensile Strength (MPa)	Elastic Modulus (GPa)	Diameter (µm)	Length (mm)
Basalt fiber	2650	4100–4800	93–110	21	6–18

**Table 5:** Results of fractal dimension of BFRCs in four regions.

	D_m_	R^2^	D_c_	R^2^	D_t_	R^2^	D_g_	R^2^
Control	2.2854	0.996	2.5078	0.998	3.0039	0.998	3.3118	0.967
BF-12-0.05%	2.3779	0.971	2.5861	0.998	2.5898	0.993	2.9989	0.978
BF-12-0.06%	2.4097	0.994	2.6134	0.999	2.7335	0.996	2.9985	0.997
BF-12-0.07%	2.4264	0.998	2.6816	0.998	2.7581	0.997	2.9938	0.998
BF-6-0.06%	2.3946	0.994	2.6622	0.999	2.7979	0.995	2.9915	0.999
BF-18-0.06%	2.4255	0.988	2.6769	0.998	2.7285	0.989	2.9827	0.994
BF-24-0.06%	2.3331	0.967	2.4719	0.989	2.6804	0.988	2.9239	0.959

**Table 6:** Correlation analysis results among pore parameters.

		FD	Porosity					D_m_	D_c_	APD	TPV	TPA
			Total	Gel	Transition	Capillary	Macro					
FD		1	0.481	0.862	0.275	0.791	−0.309	0.952 (**)	0.865	−0.937 (**)	−0.014	0.657
Porosity	Total	0.481	1	0.354	−0.502	0.350	0.601	0.383	0.189	−0.357	−0.780	0.228
	Gel	0.862	0.354	1	0.293	0.884 (**)	−0.501	0.789	0.887 (**)	−0.693	−0.251	0.717
	Transition	0.275	−0.502	0.293	1	0.250	−0.773	0.439	0.527	−0.283	−0.636	0.007
	Capillary	0.791	0.350	0.884 (**)	0.250	1	−0.507	0.754	0.933 (**)	−0.571	−0.127	0.486
	Macro	−0.309	0.601	−0.501	−0.773	−0.507	1	−0.772	−0.652	0.253	0.866 (**)	−0.282
D_m_		0.952 (**)	0.383	0.789	0.439	0.754	−0.372	1	0.891 (**)	−0.924 (**)	−0.001	0.619
D_c_		0.865	0.189	0.887 (**)	0.527	0.933 (**)	−0.652	0.891 (**)	1	−0.727	−0.269	0.542
APD		−0.937 (**)	−0.357	−0.693	−0.283	−0.571	0.253	−0.924 (**)	−0.727	1	0.013	−0.704
TPV		−0.014	0.780	−0.251	−0.636	−0.127	0.866 (**)	−0.001	−0.269	0.013	1	−0.142
TPA		0.657	0.228	0.717	0.007	0.486	−0.282	0.619	0.542	−0.704	−0.142	1

(**) Represents that the results of correlation test at the significant level of 0.01 is remarkable.

**Table 7:** Fracture parameters of BFRCs.

Samples	W_0/_(N/mm)	mg/(kg)	δ/(mm)	B × (h-a_0_)/(mm^2^)	G_f_ (N/mm)
Control	914.099	9.694	2.565	8000	751.17
BF-12-0.05%	1144.184	9.814	2.731	8000	936.79
BF-12-0.06%	1345.514	9.753	3.002	8000	1099.83
BF-12-0.07%	1409.628	9.796	3.193	8000	1152.73
BF-6-0.06%	1083.248	9.653	2.771	8000	888.01
BF-18-0.06%	1618.703	9.837	3.114	8000	1319.47
BF-24-0.06%	1077.607	9.749	2.895	8000	884.66

**Table 8:** Results of average correlation coefficient.

	FD	Porosity					D_m_	D_c_	APV	APA
		Total	Gel	Transition	Capillary	Macro				
Flexural Strength	0.895	0.757	0.746	0.865	0.708	0.772	0.971	0.774	0.625	0.828
Compressive Strength	0.862	0.752	0.720	0.759	0.691	0.831	0.992	0.691	0.629	0.818
Fracture Energy	0.818	0.687	0.772	0.844	0.772	0.803	0.889	0.612	0.634	0.868
Shrinkage Strain (28d)	0.589	0.441	0.565	0.674	0.622	0.336	0.441	0.641	0.536	0.421
